# Frailty modifies the association between opioid use and mortality in chronic kidney disease patients with diabetes: a population-based cohort study

**DOI:** 10.18632/aging.103978

**Published:** 2020-11-07

**Authors:** Szu-Ying Lee, Jui Wang, Chia-Ter Chao, Kuo-Liong Chien, Jenq-Wen Huang

**Affiliations:** 1 Department of Internal Medicine, National Taiwan University Hospital Yunlin Branch, Yunlin County, Taiwan; 2 Institute of Epidemiology and Preventive Medicine, College of Public Health, National Taiwan University, Taipei, Taiwan; 3 Nephrology Division, Department of Internal Medicine, National Taiwan University Hospital BeiHu Branch, Taipei, Taiwan; 4 Geriatric and Community Medicine Research Center, National Taiwan University Hospital BeiHu Branch, Taipei, Taiwan; 5 Graduate Institute of Toxicology, National Taiwan University College of Medicine, Taipei, Taiwan

**Keywords:** chronic kidney disease, diabetes mellitus, frail phenotype, FRAIL scale, opioid

## Abstract

The prevalence of chronic pain in patients with chronic kidney disease (CKD) and diabetes mellitus is high and correlates with higher frailty risk, but satisfactory pain control frequently fails, necessitating opioid initiation. We aimed to examine whether opioid use affected their outcomes and whether such a relationship was modified by frailty. From the longitudinal cohort of diabetes patients (n = 840,000), we identified opioid users with CKD (n = 26,029) and propensity score-matched them to opioid-naïve patients in a 1:1 ratio. We analyzed the associations between opioid use and long-term mortality according to baseline frailty status, defined by the modified FRAIL scale. Among all, 20.3% did not have any FRAIL items, while 57.2%, 20.6%, and 1.9% had 1, 2, and at least 3 positive FRAIL items, respectively. After 4.2 years, 16.4% died. Cox proportional hazard regression showed that opioid users exhibited an 18% higher mortality risk (HR 1.183, 95% CI 1.13-1.24) with a dose- and duration-responsive relationship, compared to opioid-naive ones. Furthermore, the mortality risk posed by opioids was observed only in CKD patients without frailty but not in those with frailty. In conclusion, opioid use increased mortality among patients with CKD, while this negative outcome influence was not observed among frail ones.

## INTRODUCTION

Pain, as the fifth vital sign, is a frequent complaint that prompts patients to seek medical advice and treatment, and chronic pain specifically refers to an unpleasant experience that lasts for more than 3 months despite the normal healing time [1]. The prevalence of chronic pain ranges between 20% and 50%, depending on the population being surveyed, associated morbidities, and types of pain under examination [2]. Besides its disabling influence, chronic pain potentially increases the risk of cardiovascular events [3] and long-term mortality [4]. The burden of chronic pain becomes more prominent among those of advanced age and those with morbidities, including chronic kidney disease (CKD) and diabetes mellitus (DM) [1]. Population-based studies showed that patients with CKD or DM had a greater prevalence of chronic pain than those without; more than 60% of patients with CKD have chronic pain [5, 6]. Furthermore, the presence of chronic pain might be predictive of subsequent renal function decline [7]. The origins of chronic pain in patients with DM or CKD include musculoskeletal degeneration, diabetic or uremic neuropathy, and delayed wound healing with a predilection for developing unhealed wounds [8]. Despite the observed detrimental influences on outcome and quality of life, chronic pain is frequently under-recognized and fails to be managed satisfactorily, especially in those with CKD.

Pharmacological pain control consists of acetaminophen, non-steroidal anti-inflammatory drugs (NSAIDs), gabapentinoids/anti-depressants, and opioids. However, the concerns of nephrotoxicity when prescribing NSAIDs for those with CKD increases with increasing severity of renal impairment, while the predominant renal excretion of gabapentinoids also discourages nephrologists from using them to achieve adequate pain control among patients with CKD [5, 8]. Opioids are no exception; the accumulation of parent compounds or their metabolites during renal failure similarly poses risk to those with CKD who received them. Experts have suggested that the therapeutic window of opioids in patients with CKD is very narrow, and a careful deliberation between the pros and cons of initiating opioids should be considered in this population [9]. Nonetheless, the use of opioids in patients with CKD is still common; it is estimated that 20% to 30% of patients with advanced CKD receive opioids for pain control, accounting for nearly half of those receiving analgesia [9]. This phenomenon can be worrisome, in light of the potentially adverse outcome influences related to opioid use and the scarcity of available evidence supporting this practice [10]. Very few studies have examined the effect of opioid use on the long-term mortality of patients with CKD.

Frailty, a geriatric syndrome resulting from cumulative health deficits over biological aging, exhibits a high incidence among those with advance age, and comorbid CKD and DM [11]. The presence of frailty potentially increases the risk of mortality, hospitalization, disability, and institutionalization among different populations [12, 13], indicating that frailty can be a potential outcome modifier in susceptible patients. Patients with DM and CKD are at a greater risk of developing frailty and chronic pain simultaneously, and chronic pain may precipitate subsequent frailty. Furthermore, the existing literature indicates that frailty may actually influence the therapeutic efficacy of disease-modifying medications and potentially introduce a higher risk of medication-related adverse events [14]. This is particularly of concern for those with DM and CKD, since they tend to have a higher prevalence of organ dysfunction and more severe symptomatology [15], especially chronic pain. Therefore, pain control in frail CKD patients with DM is important, for which opioids are frequently prescribed to optimize their quality of life. However, based on the current literature, whether opioids introduce risk for mortality among patients with DM and CKD remains controversial, and whether frailty modifies such risk is unknown. We hypothesized that frailty could potentially modify the relationship between opioid use and mortality among patients with DM and CKD. To answer this question, we utilized a large cohort of patients with DM and CKD to test our hypothesis.

## RESULTS

**Figure 1:**
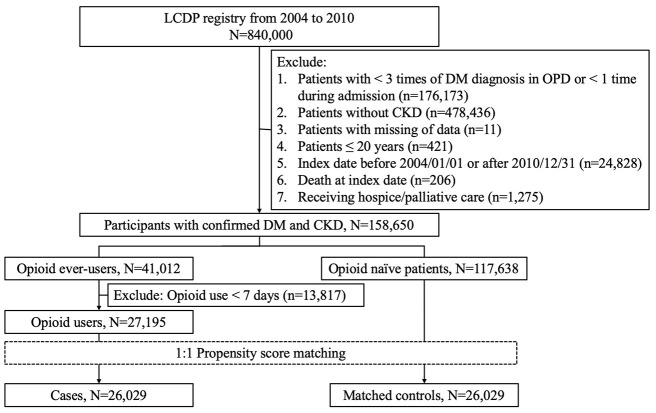
**The selection and analytic workflow of study participants from the longitudinal cohort of diabetes patients (LCDPs).** CKD, chronic kidney disease; DM, diabetes mellitus; OPD, outpatient department.

A total of 840,000 participants were included initially; after applying the exclusion criteria described above, 158,650 participants with CKD and DM remained in the analytic workflow. Among them, 117,638 (74.1%) were opioid-naïve patients during the entire study period, while 41,012 (25.9%) had prescription records of opioids (Figure 1). We further selected 26,029 cases with cumulative opioid use for ≥7 days and 26,029 propensity score-matched controls for subsequent analysis. The mean age of the opioid users and naïve patients was 62.9 years, with a nearly 1:1 male to female ratio (Table 1). The most common comorbidity among enrollees was hypertension, followed by hyperlipidemia, osteoarthritis, and chronic liver disease; the mean CCI scores and the severity of diabetes were similar to those of age-matched patients with DM in other studies [16]. The severity of CKD among enrollees was modest, as only 5% to 6% of them had stage 5 CKD (Table 1). Most medications were balanced between opioid users and naïve patients.

**Table 1:** Clinical features of opioid users and matched opioid naïve patients.

	**Opioid users (n = 26,029)**	**Naïve patients (n = 26,029)**	***p-value***
*Demographic profile*			
**Age (years)**	62.9 ± 13.3	62.9 ± 13.9	*0.934*
**Sex (Female)**	12,607 (48.3)	12,487 (48.0)	*0.293*
*Lifestyle factors*			
**Smoking (%)**	208 (0.8)	208 (0.8)	*1*
**Alcoholism (%)**	558 (2.1)	537 (2.1)	*0.521*
**Obesity (%)**	517 (2)	519 (2)	*0.95*
*CCI*	*3.9 ± 2.3*	*3.9 ± 2.3*	*0.462*
*Comorbidities*			
**Hypertension (%)**	19,657 (75.5)	19,561 (75.2)	*0.329*
Diabetic severity*	1 ± 1.3	0.99 ± 1.29	*0.367*
**Hyperlipidemia (%)**	13,871 (53.3)	13,798 (53.0)	*0.521*
**Acute coronary syndrome (%)**	9,327 (35.8)	9,301 (35.7)	*0.812*
**Atrial fibrillation (%)**	5,066 (19.5)	4,960 (19.1)	*0.239*
**Peripheral vascular disease (%)**	1,125 (4.3)	1,123 (4.3)	*0.966*
**Cerebrovascular disease (%)**	6,200 (23.8)	6,101 (23.4)	*0.307*
**Heart failure (%)**	3,865 (14.9)	3,795 (14.6)	*0.387*
**Chronic obstructive pulmonary disease (%)**	6,001 (23.1)	5,952 (22.9)	*0.610*
**Stage 5 CKD (%)**	1,541 (5.9)	1,552 (6)	*0.838*
**Chronic liver disease (%)**	11,525 (44.3)	11,504 (44.2)	*0.853*
**Malignancy (%)**	3,538 (13.6)	3,612 (13.9)	*0.346*
**Parkinsonism (%)**	685 (2.6)	710 (2.7)	*0.498*
**Osteoarthritis (any site) (%)**	13,930 (53.5)	13,866 (53.3)	*0.574*
**Gout (%)**	8,940 (34.4)	8,815 (33.9)	*0.248*
**Mental disorders (%)**	7,880 (30.3)	7,802 (30.0)	*0.456*
*Medications with outcome influences*			
**ACEi (%)**	10,538 (40.5)	10,496 (40.3)	*0.708*
**ARB (%)**	14,388 (55.3)	14,323 (55.0)	*0.567*
**β-blockers (%)**	15,706 (60.3)	15,718 (60.4)	*0.914*
**Aspirin (%)**	13,012 (50.0)	13,010 (50.0)	*0.986*
**Clopidogrel (%)**	3,075 (11.8)	2,999 (11.5)	*0.300*
**Warfarin (%)**	1,161 (4.5)	1,158 (4.5)	*0.949*
**Statin (%)**	12,435 (47.8)	12,465 (47.9)	*0.792*
**Fibrate (%)**	5,703 (21.9)	5,759 (22.1)	*0.554*
**Allopurinol (%)**	2,225 (8.6)	2,282 (8.8)	*0.374*
**NSAID (%)**	25,277 (97.1)	25,385 (97.5)	*0.003*
**COX-II inhibitor (%)**	13,819 (53.1)	13,834 (53.2)	*0.895*
**Anti-depressants (%)**	9,686 (37.2)	9,635 (37.0)	*0.644*
**Anti-psychotics (%)**	10,954 (42.1)	10,846 (41.7)	*0.337*
**Benzodiazepine (%)**	19,273 (74.0)	19,269 (74.0)	*0.968*
*Anti-diabetic agents*			
**Sulfonylurea (%)**	13,378 (51.4)	13,608 (52.3)	*0.043*
**Biguanide (%)**	14,199 (54.6)	14,359 (55.2)	*0.159*
**Insulin (%)**	4,531 (17.4)	4,428 (17.0)	*0.232*
**α-glucosidase inhibitor (%)**	4,674 (18.0)	4,695 (18.0)	*0.811*
**Meglitinide (%)**	3,952 (15.2)	3,995 (15.4)	*0.600*
**Thiazolidinedione (%)**	2,868 (11.0)	2,826 (10.9)	*0.555*
**DPP4 inhibitors (%)**	2,746 (10.6)	2,690 (10.3)	*0.422*
*Treatment variables*			
**Coronary revascularization (%)**	410 (1.6)	402 (1.5)	*0.777*
**Cardiac surgery (any) (%)**	758 (2.9)	735 (2.8)	*0.546*
**ICU stay (%)**	2,375 (9.1)	2,308 (8.9)	*0.305*
*Number of FRAIL items*			*0.470*
**0**	5,270 (20.3)	5,303 (20.4)	
**1**	14,827 (57.0)	14,947 (57.4)	
**2**	5,451 (20.9)	5,291 (20.3)	
**3**	453 (1.7)	454 (1.7)	
**4**	28 (0.1)	34 (0.1)	
*Follow-up durations*			
**Median (IQR) (years)**	4.26 (3.39)	4.22 (3.38)	*0.260*
**Minimum / Maximum (years)**	0.0027 / 8.0	0.0027 / 8.0	*NA*

*Based on adapted diabetes complications severity index (aDCSI)

NA, not applicable

ACEi, angiotensin-converting enzyme inhibitor; ARB, angiotensin receptor blocker; CCI, Charlson comorbidity index; CKD, chronic kidney disease; COX, cyclo-oxygenase; DPP4, dipeptidyl peptidase 4; ICU, intensive care unit; IQR, interquartile range; NSAID, non-steroidal anti-inflammatory agent

Among all enrollees, more than half had osteoarthritis (53%), one-third had gout (34%), and only 14% had malignancies (Table 1). Since we already excluded those receiving hospice/palliative care, it is likely that these patients mainly received opioids to manage their musculoskeletal disorders and/or diabetic neuropathy-related pain [17]. This is supported by our findings that a very high proportion of users and non-users also received NSAIDs (~97%) (Table 1).

Among 52,058 enrollees with DM and CKD, 969 (1.86%) had frailty (FRAIL item count ≥ 3), while 10,573 (20.31%), 29,774 (57.19%), and 10,742 (20.63%) did not have any, had 1, and had 2 positive FRAIL items, respectively. There was no significant difference in the proportion of frailty between opioid users and naïve patients (1.85% vs. 1.87%). Importantly, there was no significant difference between opioid users and naïve patients with regard to the number of FRAIL items at baseline (*p* = 0.47) (Table 1). The most common FRAIL item present was “illness” (75%), followed by “fatigue” (24%) and “loss of weight” (2.5%) (Table 2). There was no significant difference regarding most FRAIL item positivity, except “illness” (users vs. naïve patients, 75.3% vs. 74%; *p* < 0.001) between opioid users and naïve patients.

**Table 2:** FRAIL item positivity based on opioid use status.

	**Opioid users (n = 26,029)**	**Naïve patients (n = 26,029)**	***p*-value**
*FRAIL item*			
**Fatigue**	6,222 (23.9)	6,382 (24.5)	*0.102*
**Resistance**	580 (2.2)	545 (2.1)	*0.291*
**Ambulation**	263 (1.0)	260 (1.0)	*0.895*
**Illness**	19,606 (75.3)	19,253 (74.0)	*<0.001*
**Loss of weight**	529 (2.0)	587 (2.3)	*0.079*

CKD, chronic kidney disease

**Figure 2:**
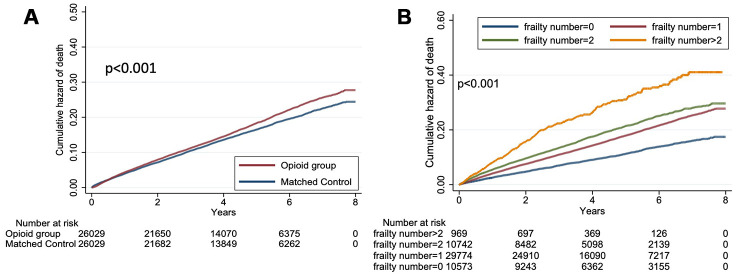
****Kaplan-Meier survival curves based on opioid use or not (**A**) and FRAIL item counts (**B**) among the total cohort (n = 52,058).

After a mean of 4.23 years of follow-up, 8,558 participants died, resulting in a mortality rate of 16.4%. There was no significant difference with regard to follow-up durations between opioid users and naïve patients (*p* = 0.26) (Table 1). Univariate analysis showed that opioid users with DM and CKD had a significantly higher mortality than opioid-naïve ones (mortality rate 17.4% vs. 15.4% for users and naïve patients, hazard ratio [HR] 1.124, 95% confidence interval [CI] 1.08 – 1.17) (Table 3). Kaplan-Meier survival analysis revealed that opioid users had a significantly increased risk of mortality during follow-up compared to opioid-naïve ones (*p* < 0.001; Figure 2A). In addition, patients with an increasing number of FRAIL items similarly had a stepwise higher mortality during follow up compared to those without any items (Figure 2B). Cox proportional hazard regression revealed that opioid users exhibited an 18.3% higher risk of mortality than opioid-naïve ones (HR 1.183, 95% CI 1.13-1.24), independent of demographic profiles, lifestyle factors, comorbidities, aDCSI, FRAIL item counts, and treatment variables (model A; Table 3). The results were essentially unchanged when individual FRAIL item status was considered (model B; Table 3). Patients with 1, 2, and 3 FRAIL items exhibited a stepwise increasing risk of mortality after adjusting for clinical variables (for those with 1, 2, and 3 items, HR 1.048, 1.18, and 1.195; 95% CI 0.97 – 1.13, 1.08 – 1.29, and 1.03 – 1.39, respectively), compared to those without any item.

**Table 3:** Cox proportional hazard regression with patient mortality as the dependent variable.

**Outcomes**	**Events**	**Person-year^#^**	Incidence density*	**Crude^$^**		**Model A^&^**		**Model B^%^**	
				**HR**	**95% CI**	**HR**	**95% CI**	**HR**	**95% CI**
*Total cohort*									
Naïve patients	4,018	110,097.3	36.5	1	-	1	-	1	-
Users	4,540	110,622.9	41.0	1.124	1.08 – 1.17^a^	1.183	1.13 – 1.24^a^	1.183	1.13 – 1.24^a^
*Frail participants (Item ≥ 3)*									
Naïve patients	137	1,646.2	83.2	1	-	1	-	1	-
Users	126	1,707.4	73.8	0.886	0.7 – 1.13	1.08	0.83 – 1.4	1.079	0.83 – 1.41
*Non-frail participants (Item < 3)*									
Naïve patients	3,881	108,451.1	35.8	1	-	1	-	1	-
Users	4,414	108,915.5	40.5	1.12	1.07 – 1.19^a^	1.183	1.13 – 1.24^a^	1.183	1.13 – 1.24^a^
*Non-frail participants (Item = 0)*									
Naïve patients	444	24,638.9	18.0	1	-	1	-	1	-
Users	743	23,874.6	31.1	1.727	1.54 – 1.94^a^	1.503	1.33 – 1.69^a^	1.503	1.33 – 1.69^a^
*Non-frail participants (Item = 1)*									
Naïve patients	2,383	63,425.7	37.6	1	-	1	-	1	-
Users	2,693	63,166.5	42.6	1.134	1.07 – 1.2^a^	1.173	1.11 – 1.24^a^	1.175	1.11 – 1.24^a^
*Non-frail participants (Item = 2)*									
Naïve patients	1,054	20,386.5	51.7	1	-	1	-	1	-
Users	978	21,874.5	44.7	0.866	0.79 – 0.95^b^	1.026	0.94 – 1.12	1.024	0.94 – 1.12

aDCSI, adapted diabetes complications severity index; CI, confidence interval; HR, hazard ratio

^#^Cumulative follow-up duration

^·^ per 1000 patient-year

^$^ Incorporating opioid use only

^&^ Incorporating age/gender, lifestyle factors, all comorbidities, aDCSI, all medications, FRAIL item counts, treatment variables, and opioid use; findings without an attached superscript “a” or “b” denote insignificant results (*p* > 0.05)

^%^ Incorporating model A components and individual FRAIL item status

^a^ *p* < 0.001

^b^ *p* < 0.01

We then examined the dose- and duration-dependent relationship between opioid use and mortality. After dividing participants into those receiving < 28 days, 28 to 84 days, and ≥ 84 days of opioid cumulatively, we found that those receiving a longer duration of opioid treatment had a significantly higher mortality compared to opioid-naïve patients (for < 28 days, 28 to 84 days, and ≥ 84 days, HR 1.113, 1.189, and 1.296; 95% CI 1.05 – 1.18, 1.12 – 1.26, and 1.22 – 1.38, respectively), independent of other clinical variables (Table 4). After dividing participants into quartiles according to their defined daily dosage (DDD) of opioid use, we similarly found that those receiving a greater DDD of opioid had a stepwise increasing risk of mortality independent of other clinical variables (Table 5).

**Table 4:** Cox proportional hazard regression analyses based on the tertiles of the duration of opioid use in the total cohort and each FRAIL item group.

**Outcomes**	**Events**	**Person-year^#^**	Incidence density*	**Crude^$^**		**Model A^&^**	
				**HR**	**95% CI**	**HR**	**95% CI**
*Total cohort*							
Naïve patients	4,018	110,097.3	36.5	1	-	1	-
< 28 days	1,833	50,416.4	36.4	0.996	0.94 – 1.05	1.113	1.05 – 1.18^b^
28 – 84 days	1,468	34,834.8	42.1	1.154	1.09 – 1.23^a^	1.189	1.12 – 1.26^a^
≥ 84 days	1,239	25,371.8	48.8	1.339	1.26 – 1.43^a^	1.296	1.22 – 1.38^a^
*Frail participants (Item ≥ 3)*							
Naïve patients	137	1,646.2	83.2	1	-	1	-
< 28 days	52	626.6	83.0	0.999	0.73 – 1.37	1.350	0.95 – 1.91
28 – 84 days	37	630.2	58.7	0.707	0.49 – 1.02	0.821	0.56 – 1.21
≥ 84 days	37	450.6	82.1	0.980	0.68 – 1.41	1.110	0.75 – 1.66
*Non-frail participants (Item = 0)*							
Naïve patients	444	24,638.9	18.0	1	-	1	-
< 28 days	340	13,947.6	25.2	1.398	1.21 – 1.61^a^	1.297	1.12 – 1.50^b^
28 – 84 days	226	6,723.2	33.6	1.864	1.59 – 2.19^a^	1.579	1.34 – 1.86^a^
≥ 84 days	177	3653.9	48.4	2.691	2.26 – 3.20^a^	1.993	1.67 – 2.38^a^
*Non-frail participants (Item = 1)*							
Naïve patients	2,383	63,425.7	37.6	1	-	1	-
< 28 days	1,050	27,533.3	38.1	1.013	0.94 – 1.09	1.090	1.01 – 1.17^c^
28 – 84 days	879	20,372.4	43.2	1.148	1.06 – 1.24^b^	1.158	1.07 – 1.25^b^
≥ 84 days	764	15,260.8	50.1	1.334	1.23 – 1.45^a^	1.329	1.22 – 1.44^a^
*Non-frail participants (Item = 2)*							
Naïve patients	1,054	20,386.5	51.7	1	-	1	-
< 28 days	391	8,758.9	44.6	0.864	0.77 – 0.97^c^	1.028	0.91 – 1.16
28 – 84 days	326	7,109.0	45.9	0.889	0.79 – 1.01	1.122	0.99 – 1.27
≥ 84 days	261	6,006.6	43.5	0.842	0.74 – 0.97^c^	0.925	0.81 – 1.06

aDCSI, adapted diabetes complications severity index; CI, confidence interval; HR, hazard ratio

^#^Cumulative follow-up duration

^·^ per 1000 patient-year

^$^ Incorporating opioid use only

^&^ Incorporating age/gender, lifestyle factors, all comorbidities, aDCSI, all medications, FRAIL item counts, treatment variables, and opioid use; findings without an attached superscript “a” or “b” denote insignificant results (*p* > 0.05)

^a^ *p* < 0.001

^b^ *p* < 0.01

^c^ *p* < 0.05

**Table 5:** Cox proportional hazard regression analyses based on the quartiles of the defined daily dosage of opioid in the total cohort and each FRAIL item group.

**Outcomes**	**Events**	**Person-year^#^**	Incidence density*	**Crude^$^**		**Model A^&^**	
				**HR**	**95% CI**	**HR**	**95% CI**
*Total cohort*							
Naïve patients	4,018	110,097.3	36.5	1	-	1	-
Quartile 1	858	28,104.3	30.5	0.837	0.78 – 0.90^a^	0.923	0.86 – 0.99^c^
Quartile 2	885	28,304.1	31.3	0.857	0.80 – 0.92^a^	0.966	0.90 – 1.04
Quartile 3	1,143	27,540.3	41.5	1.137	1.07 – 1.21^b^	1.196	1.12 - 1.28^a^
Quartile 4	1,654	26,674.3	62.0	1.698	1.60 – 1.80^a^	1.592	1.50 – 1.69^a^
*Frail participants (Item ≥ 3)*							
Naïve patients	137	1,646.2	83.2	1	-	1	-
Quartile 1	27	348.3	77.5	0.926	0.61 – 1.40	1.198	0.77 – 1.88
Quartile 2	25	404.6	61.8	0.744	0.49 – 1.14	0.817	0.52 – 1.28
Quartile 3	29	434.1	66.8	0.805	0.54 – 1.20	1.039	0.67 – 1.61
Quartile 4	45	520.4	86.5	1.036	0.74 – 1.45	1.282	0.88 – 1.86
*Non-frail participants (Item = 0)*							
Naïve patients	444	24,638.9	18.0	1	-	1	-
Quartile 1	157	8290.6	18.9	1.052	0.88 – 1.26	0.964	0.80 – 1.16
Quartile 2	159	6324.2	25.1	1.395	1.16 – 1.67^b^	1.304	1.09 – 1.57^b^
Quartile 3	164	5083.2	32.3	1.79	1.50 – 2.14^a^	1.603	1.34 – 1.92^a^
Quartile 4	263	4176.7	63.0	3.493	3.00 – 4.07^a^	2.528	2.16 – 2.96^a^
*Non-frail participants (Item = 1)*							
Naïve patients	2,383	63,425.7	37.6	1	-	1	-
Quartile 1	481	14,932.3	32.2	0.856	0.78 – 0.95^b^	0.873	0.79 – 0.96^b^
Quartile 2	531	16,261.8	32.7	0.869	0.79 – 0.95^b^	0.972	0.88 – 1.07
Quartile 3	680	16,239.0	41.9	1.114	1.02 – 1.21^c^	1.179	1.08 – 1.29^b^
Quartile 4	1,001	15,733.3	63.6	1.693	1.57 – 1.82^a^	1.602	1.49 – 1.73^a^
*Non-frail participants (Item = 2)*							
Naïve patients	1,054	20,386.5	51.7	1	-	1	-
Quartile 1	193	4,533.2	42.6	0.824	0.71 – 0.96^c^	1.048	0.90 – 1.22
Quartile 2	170	5,313.4	32.0	0.621	0.53 – 0.73^a^	0.764	0.65 – 0.90^b^
Quartile 3	270	5,784.1	46.7	0.904	0.79 – 1.03	1.070	0.94 – 1.23
Quartile 4	345	6,243.9	55.3	1.071	0.95 – 1.21	1.174	1.04 – 1.33^c^

aDCSI, adapted diabetes complications severity index; CI, confidence interval; HR, hazard ratio

^#^Cumulative follow-up duration

^·^ per 1000 patient-year

^$^ Incorporating opioid use only

^&^ Incorporating age/gender, lifestyle factors, all comorbidities, aDCSI, all medications, FRAIL item counts, treatment variables, and opioid use; findings without an attached superscript “a” or “b” denote insignificant results (*p* > 0.05)

^a^ *p* < 0.001

^b^ *p* < 0.01

^c^ *p* < 0.05

**Figure 3:**
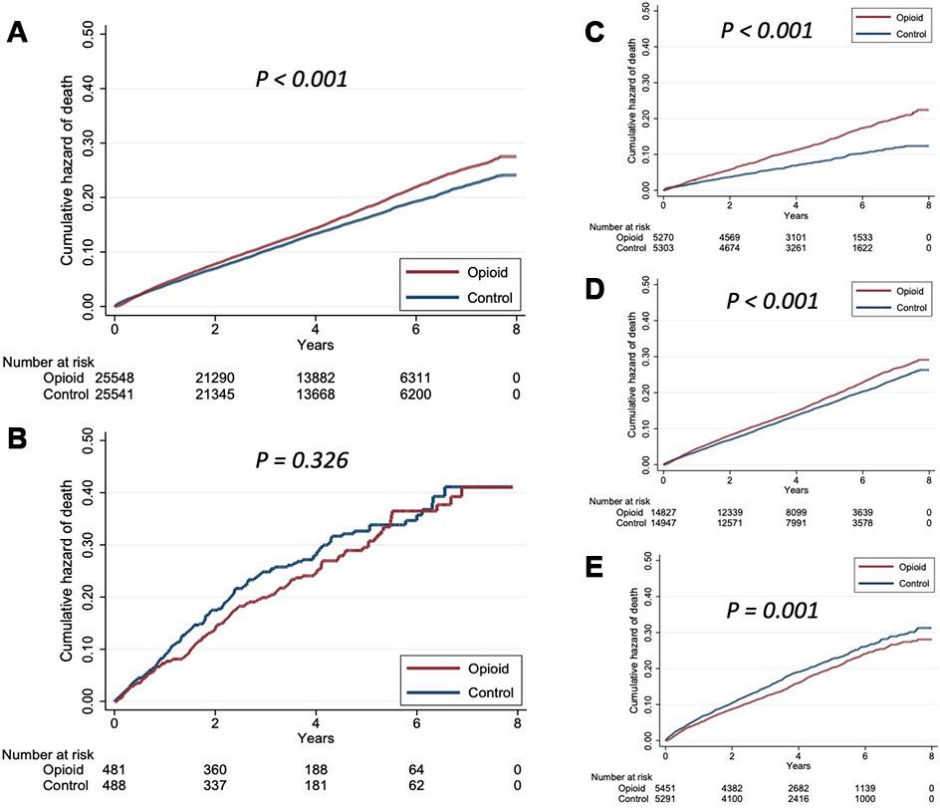
****Kaplan-Meier survival curves based on (**A**) frailty presence; (**B**) frailty absence; (**C**) having none of the FRAIL items; (**D**) having 1 positive FRAIL item; and (**E**) having 2 positive FRAIL items.

We subsequently categorized participants according to their baseline FRAIL item counts. Baseline characteristics between opioid users and naïve patients were mostly balanced among those without and with 1, 2, and >2 FRAIL items (Table 6). Interestingly, we discovered that the increased mortality risk conferred by opioid use was present only among those without frailty (HR 1.183, 95% CI 1.13-1.24), but not among those with frailty (FRAIL item ≥ 3) (HR 1.08, 95% CI 0.83 – 1.4) (model A in Table 3; Figure 3A and 3B), independent of other clinical variables. We further examined such relationships among those without any FRAIL items, with 1 and 2 positive FRAIL items. Kaplan-Meier survival analyses revealed that opioid users without (Figure 3C) and with 1 FRAIL item (Figure 3D) still had a higher mortality risk than opioid-naïve ones; however, the risk was not observed in those with 2 FRAIL items (Figure 3E). The risk brought by opioid use among non-frail participants decreased with increasing numbers of positive FRAIL items (for FRAIL items 0, 1, and 2, HR 1.503, 1.173, and 1.026, 95% CI 1.33 – 1.69, 1.11 – 1.24, and 0.94 – 1.12, respectively) (model A in Table 3). The duration-dependent and dosage-dependent relationship between opioid use and mortality was also attenuated gradually when the number of positive FRAIL items increased (Tables 4 and 5). These findings suggest that the association between opioid use and increased mortality might alter depending on the severity of frailty, and the association could be insignificant among those with full-fledged frailty (FRAIL item ≥ 3).

**Table 6:** Clinical features of opioid users and naïve patients based on their baseline FRAIL item counts.

	**FRAIL item = 0**			**FRAIL item = 1**			**FRAIL item = 2**			**FRAIL item > 2**		
	**Users (n = 5,270)**	**Naïve (n = 5,303)**	***p-value***	**Users (n = 14,827)**	**Naïve (n = 14,947)**	***p-value***	**Users (n = 5,451)**	**Naïve (n = 5,291)**	***p-value***	**Users (n = 481)**	**Naïve (n = 488)**	***p-value***
*Demographic profile*												
**Age (years)**	53.6 ± 12.4	53.0 ± 13.0	*0.009*	64.5 ± 12.5	64.5 ± 12.9	*0.719*	66.6 ± 12.4	67.4 ± 12.7	*0.002*	70.7 ± 12.2	71.9 ± 12.0	*0.133*
**Sex (Female)**	2070 (39.3)	2301 (43.4)	*<0.001*	*7,308 (49.3)*	*7,178 (48.0)*	*0.029*	*2,978 (54.6)*	*2,768 (52.3)*	*0.016*	*251 (52.2)*	*240 (49.2)*	*0.350*
*Lifestyle factors*												
**Smoking (%)**	34 (0.7)	33 (0.6)	*0.882*	115 (0.8)	116 (0.8)	*0.996*	57 (1.1)	51 (1.0)	*0.671*	2 (0.4)	8 (1.6)	*0.060*
**Alcoholism (%)**	188 (3.6)	140 (2.6)	*0.006*	252 (1.7)	255 (1.7)	*0.966*	107 (2.0)	118 (2.2)	*0.334*	11 (2.3)	24 (4.9)	*0.028*
**Obesity (%)**	100 (1.9)	128 (2.4)	*0.068*	290 (2.0)	268 (1.8)	*0.300*	120 (2.2)	117 (2.2)	*0.972*	7 (1.5)	6 (1.2)	*0.760*
*CCI*	2.5 ± 1.7	2.5 ± 1.5	*0.01*	4.1 ± 2.3	4.1 ± 2.2	*0.594*	4.7 ± 2.4	4.7 ± 2.4	*0.188*	5.5 ± 2.7	5.6 ± 2.5	*0.482*
*Comorbidities*												
**Hypertension (%)**	1,759 (33.4)	1,814 (34.2)	*0.367*	12,621 (85.1)	12,614 (84.4)	*0.080*	4,846 (88.9)	4,690 (88.6)	*0.670*	431 (89.6)	443 (90.8)	*0.539*
Diabetic severity*	0.5 ± 0.9	0.5 ± 0.9	*0.751*	1.1 ± 1.3	1.0 ± 1.3	*0.140*	1.2 ± 1.4	1.3 ± 1.4	*0.606*	1.5 ± 1.5	1.7 ± 1.6	*0.159*
**Hyperlipidemia (%)**	2,171 (41.2)	2,341 (44.1)	*0.002*	8,157 (55.0)	8,126 (54.4)	*0.261*	3,279 (60.2)	3,082 (58.3)	*0.045*	264 (54.9)	249 (51.0)	*0.229*
**Acute coronary syndrome (%)**	455 (8.6)	497 (9.4)	*0.185*	5,938 (40.1)	5,959 (39.9)	*0.750*	2,678 (49.1)	2,555 (48.3)	*0.384*	256 (53.2)	290 (59.4)	*0.052*
**Atrial fibrillation (%)**	287 (5.5)	304 (5.7)	*0.521*	3,084 (20.8)	3,057 (20.5)	*0.459*	1,511 (27.7)	1,438 (27.2)	*0.530*	184 (38.3)	161 (33.0)	*0.087*
**Peripheral vascular disease (%)**	83 (1.6)	96 (1.8)	*0.348*	683 (4.6)	634 (4.2)	*0.126*	329 (6.0)	353 (6.7)	*0.177*	30 (6.2)	40 (8.2)	*0.239*
**Cerebrovascular disease (%)**	56 (1.1)	67 (1.3)	*0.336*	4,003 (27.0)	3,973 (26.6)	*0.416*	1,923 (35.3)	1,814 (34.3)	*0.280*	218 (45.3)	247 (50.6)	*0.099*
**Heart failure (%)**	27 (0.5)	39 (0.7)	*0.145*	2,601 (17.5)	2,485 (16.6)	*0.036*	1,104 (20.3)	1,147 (21.7)	*0.070*	133 (27.7)	124 (25.4)	*0.430*
**Chronic obstructive pulmonary disease (%)**	82 (1.6)	86 (1.6)	*0.787*	3,842 (25.9)	3,844 (25.7)	*0.701*	1,869 (34.3)	1,799 (34.0)	*0.755*	208 (43.2)	223 (45.7)	*0.442*
**Stage 5 CKD (%)**	415 (7.9)	387 (7.3)	*0.263*	889 (6.0)	901 (6.0)	*0.907*	226 (4.2)	245 (4.6)	*0.220*	11 (2.3)	19 (3.9)	*0.149*
**Chronic liver disease (%)**	2,000 (38.0)	1,980 (37.3)	*0.515*	6,330 (42.7)	6,418 (42.9)	*0.668*	2,916 (53.5)	2,824 (53.4)	*0.900*	279 (58.0)	282 (57.8)	*0.945*
**Malignancy (%)**	271 (5.1)	152 (2.9)	*<0.001*	2,291 (15.5)	2,362 (15.8)	*0.404*	885 (16.2)	980 (18.5)	*0.002*	91 (18.9)	118 (24.2)	*0.047*
**Parkinsonism (%)**	31 (0.6)	27 (0.5)	*0.582*	382 (2.6)	384 (2.6)	*0.968*	236 (4.3)	245 (4.6)	*0.451*	36 (7.5)	54 (11.1)	*0.055*
**Osteoarthritis (any site) (%)**	1,280 (24.3)	1,276 (24.1)	*0.786*	8,442 (56.9)	8,542 (57.2)	*0.712*	3,815 (70.0_	3,657 (69.1)	*0.327*	393 (81.7)	391 (80.1)	*0.531*
**Gout (%)**	1,059 (20.1)	1,073 (20.2)	*0.859*	5,432 (36.6)	5,396 (36.1)	*0.337*	2,252 (41.3)	2,135 (40.4)	*0.311*	197 (41.0)	211 (43.2)	*0.472*
**Mental disorders (%)**	821 (15.6)	863 (16.3)	*0.329*	4,446 (30.0)	4,485 (30.0)	*0.970*	2,366 (43.4)	2,207 (41.7)	*0.076*	247 (51.4)	247 (50.6)	*0.819*
*Medications with outcome influences*												
**ACEi (%)**	1,636 (31.0)	1,707 (32.2)	*0.205*	6,351 (42.8)	6,432 (43.0)	*0.730*	2,346 (43.0)	2,170 (41.0)	*0.034*	205 (42.6)	187 (38.3)	*0.173*
**ARB (%)**	2,060 (39.1)	2,188 (41.3)	*0.023*	8,812 (59.4)	8,898 (59.5)	*0.863*	3,249 (59.6)	2,989 (56.5)	*0.001*	267 (55.5)	248 (50.8)	*0.144*
**β-blockers (%)**	2,388 (45.3)	2,458 (46.4)	*0.284*	9,427 (63.6)	9,554 (63.9)	*0.543*	3,599 (66.0)	3,415 (64.5)	*0.107*	292 (60.7)	291 (59.6)	*0.732*
**Aspirin (%)**	1,806 (34.3)	1,817 (34.3)	*0.995*	7,880 (53.2)	8,072 (54.0)	*0.138*	3,053 (56.0)	2,855 (54.0)	*0.033*	273 (56.8)	266 (54.5)	*0.481*
**Clopidogrel (%)**	310 (5.9)	317 (6.0)	*0.836*	2,001 (13.5)	1,956 (13.1)	*0.298*	708 (13.0)	665 (12.6)	*0.515*	56 (11.6)	61 (12.5)	*0.682*
**Warfarin (%)**	114 (2.2)	119 (2.2)	*0.777*	759 (5.1)	736 (4.9)	*0.441*	257 (4.7)	281 (5.3)	*0.157*	31 (6.4)	22 (4.5)	*0.185*
**Statin (%)**	2,398 (45.5)	2,530 (47.7)	*0.023*	7,296 (49.2)	7,367 (49.3)	*0.890*	2,553 (46.8)	2,403 (45.4)	*0.140*	188 (39.1)	165 (33.8)	*0.089*
**Fibrate (%)**	1,229 (23.3)	1,251 (23.6)	*0.744*	3,260 (22.0)	3,303 (22.1)	*0.817*	1,141 (20.9)	1,123 (21.2)	*0.710*	73 (15.2)	82 (16.8)	*0.490*
**Allopurinol (%)**	288 (5.5)	306 (5.8)	*0.495*	1,353 (9.1)	1,435 (9.6)	*0.159*	531 (9.7)	493 (9.3)	*0.455*	53 (11.0)	48 (9.8)	*0.547*
**NSAID (%)**	5,109 (96.9)	5,160 (97.3)	*0.270*	14,376 (97.0)	14,607 (97.7)	*< 0.001*	5,324 (97.7)	5,148 (97.3)	*0.217*	468 (97.3)	470 (96.3)	*0.383*
**COX-II inhibitor (%)**	1,801 (34.2)	1,887 (35.6)	*0.129*	8,251 (55.7)	8,415 (56.3)	*0.258*	3,464 (63.6)	3,249 (61.4)	*0.022*	303 (63.0)	283 (58.0)	*0.111*
**Anti-depressants (%)**	1,585 (30.1)	1,594 (30.1)	*0.984*	5,517 (37.2)	5,527 (37.0)	*0.679*	2,367 (43.4)	2,292 (43.3)	*0.913*	217 (45.1)	222 (45.5)	*0.906*
**Anti-psychotics (%)**	1,803 (34.2)	1,837 (34.6)	*0.643*	6,190 (41.8)	6,242 (41.8)	*0.982*	2,692 (49.4)	2,522 (47.7)	*0.075*	269 (55.9)	245 (50.2)	*0.074*
**Benzodiazepine (%)**	3,378 (64.1)	3,461 (65.3)	*0.210*	11,086 (74.8)	11,205 (75.0)	*0.697*	4,437 (81.4)	4,223 (79.8)	*0.038*	372 (77.3)	380 (77.9)	*0.843*
*Anti-diabetic agents*												
**Sulfonylurea (%)**	3,210 (60.9)	3,292 (62.1)	*0.218*	7,471 (50.4)	7,680 (51.4)	*0.086*	2,523 (46.3)	2,470 (46.7)	*0.679*	174 (36.2)	166 (34.0)	*0.482*
**Biguanide (%)**	3,373 (64.0)	3,389 (63.9)	*0.918*	7,887 (53.2)	8,152 (54.5)	*0.020*	2,734 (50.2)	2,650 (50.1)	*0.941*	205 (42.6)	168 (34.4)	*0.009*
**Insulin (%)**	1,189 (22.6)	1,164 (22.0)	*0.450*	2,443 (16.5)	2,390 (16.0)	*0.255*	836 (15.3)	813 (15.4)	*0.967*	63 (13.1)	61 (12.5)	*0.781*
**α-glucosidase inhibitor (%)**	1,135 (21.5)	1,121 (21.1)	*0.617*	2,643 (17.8)	2,685 (18.0)	*0.756*	836 (15.3)	837 (15.8)	*0.490*	60 (12.5)	52 (10.7)	*0.376*
**Meglitinide (%)**	889 (16.9)	930 (17.5)	*0.363*	2,265 (15.3)	2,272 (15.2)	*0.856*	744 (13.7)	748 (14.1)	*0.464*	54 (11.2)	45 (9.2)	*0.303*
**Thiazolidinedione (%)**	871 (16.5)	800 (15.1)	*0.042*	1,497 (10.1)	1,572 (10.5)	*0.233*	465 (8.5)	433 (8.2)	*0.516*	35 (7.3)	21 (4.3)	*0.047*
**DPP4 inhibitors (%)**	748 (14.2)	725 (13.7)	*0.438*	1,511 (10.2)	1,503 (10.1)	*0.699*	461 (8.5)	440 (8.3)	*0.792*	26 (5.4)	22 (4.5)	*0.520*
*Treatment variables*												
**Coronary revascularization (%)**	9 (0.2)	22 (0.4)	*0.020*	300 (2.0)	283 (1.9)	*0.418*	93 (1.7)	93 (1.8)	*0.838*	8 (1.7)	4 (0.8)	*0.235*
**Cardiac surgery (any) (%)**	20 (0.4)	33 (0.6)	*0.077*	532 (3.6)	517 (3.5)	*0.546*	189 (3.5)	175 (3.3)	*0.647*	17 (3.5)	10 (2.1)	*0.160*

* Based on adapted diabetes complications severity index (aDCSI)

ACEi, angiotensin-converting enzyme inhibitor; ARB, angiotensin receptor blocker; CCI, Charlson comorbidity index; CKD, chronic kidney disease; COX, cyclo-oxygenase; DPP4, dipeptidyl peptidase 4; ICU, intensive care unit; NSAID, non-steroidal anti-inflammatory agent

## DISCUSSION

In this study, we examined the risk of mortality associated with opioid use in a representative population-based cohort of patients with DM and CKD, which were at a greater risk of developing opioid-related side effects. Those receiving opioids exhibited an increased risk of mortality compared to opioid-naïve patients; furthermore, the risk of mortality was modified by frailty status. Even among those who were deemed non-frail, the risk associated with opioid use might change according to their frail severity. Based on our findings, a nondiscriminatory avoidance of opioid use in patients with DM and CKD may not be appropriate; opioid use might not be associated with an increased mortality in frail patients with CKD and thus can be a viable option for pain control.

Chronic pain is common among patients with end-stage renal disease (ESRD) and advanced CKD. An international survey of analgesic prescriptions by the Dialysis Outcomes and Practice Patterns Study (DOPPS) disclosed that up to one third of ESRD patients received analgesia, among whom less than half were low potency narcotics [18]. More importantly, nearly three-fourths of ESRD patients reporting pain that compromised their working ability receive no analgesics, suggesting that pain control is unsatisfactory in these patients [18]. Inadequate pain management among patients with CKD is frequently due to the increased risk of adverse effects from opioid use, the physicians’ and patients’ concern of polypharmacy, and insufficient awareness and training regarding analgesia among nephrologists [8, 19]. However, opioid use in patients with CKD is widely known to increase side effects occurrence compared to those without, due to their altered pharmacokinetics, rising probability of drug-drug interactions, and multimorbidity [20]. A recent study reported that opioid users had a 70% higher mortality risk compared to matched NSAID users with eGFR at 80 ml/min/1.73m^2^, while the risk was elevated to more than 3-fold when their eGFR reached 40 ml/min/1.73m^2^ [21]. To overcome these barriers, it is imperative to identify the subpopulation of patients with CKD that can be immune to opioid-related adverse outcome influences. We believe that frail CKD patients can be among the candidates suitable for receiving opioids as their analgesia of choice.

The demographic features and clinical characteristics of opioid users in this study differ in several aspects from those of other populations with similar morbidities (CKD or DM) in the literature. The mean age of opioid users, especially chronic users, tended to be lower in countries other than ours. For example, a registry-based study from the United States identified a mean age of chronic opioid users at 54 to 57 years [21], while that in this study was substantially older (mean 62.9 years; Table 1). In addition, opioid users in this study were less likely to have harmful habits (alcoholism and smoking) and more likely to have morbidities such as cardiovascular diseases than those from other reports [21]. These differences reflect the diverse opioid prescription patterns observed between Western and Asian countries, and may partially account for the variations in risk factors for opioid-related side effects.

The association between opioid use and mortality in patients with CKD is rarely addressed, and for those who focus on this topic, the study design is usually suboptimal and of low quality. A recent systematic review emphasized the lack of sufficient evidence to support or refute opioid use in patients with CKD, and available studies contained low sample sizes and addressed side effects but not mortality with prominent variations in opioid types and the route of administration [10]. Oh et al. reported that chronic opioid use was not associated with a higher risk of mortality among those with CKD from 2990 critically ill patients [22]. Another Korean study reported that the association between chronic opioid use and mortality in the general population differed depending on opioid potency; those with a strong potency predominantly contributed to the observed mortality risk [23]. However, existing studies rarely focus on the CKD population, and the extrapolation of findings from critically ill, surgical, or general populations to those with CKD can be inaccurate. Our findings are expected to fill this knowledge gap by showing that opioid use increased mortality in patients with CKD, independent of multiple confounders (Table 3).

The modification effect of frailty on the outcome association of opioids has not been observed in patients with CKD but has been observed in other populations. Kim et al. analyzed Medicare enrollees with osteoarthritis undergoing total knee replacement and discovered that opioid users had a significantly higher risk of operation revisions and mortality than naïve patients; however, when comorbidities and frailty scores were adjusted for, the associations between opioid use and the elevated mortality disappeared [24]. Several reasons may be responsible for this effect introduced by frailty. First, being frail places patients with CKD in an accelerated aging process, increasing their long-term mortality, potentially diminishing the mortality differences introduced by opioid use. In addition, frailty often co-exists with other morbidities, especially in those with CKD, including cardiovascular diseases, chronic inflammation, and sarcopenia, all of which may partially account for the detrimental influences of opioids [25]. Consequently, for these frail CKD patients, opioid use may not be as harmful as for non-frail patients.

Our study has several strengths and limitations. The validity of our findings is greatly enhanced by our population-based cohort, the large sample size, and the extensive adjustment of outcome-influencing variables. Imbalances between opioid users and opioid-naïve patients regarding clinical features are unlikely. The lower risk of mortality caused by opioids over rising frailty severity also supports the validity of our findings. However, several limitations remain to be noted. First, we cannot differentiate between those who intermittently used and continuously used opioids; there are studies suggesting that outcomes differ between continuous and intermittent opioid users [24]. If we restrict analyses to those who received opioids for continuously longer than 7 days, the number of included patients may diminish meaningfully, compromising the statistical efficiency for detecting outcome influences. This is due to the fact that opioid prescription remains an uncommon practice in Taiwan compared to that in other developed countries [26]. In addition, we did not examine other side effects of opioids such as gastrointestinal, cognitive, and respiratory symptoms; therefore, we cannot derive any conclusion regarding the risk of opioid-related side effects among patients with CKD. More evidence is needed to affirm and extend our results.

In conclusion, we conducted a population-based study to examine the relationship between opioid use and mortality among patients with DM and CKD. We discovered that opioid use was associated with a higher risk of mortality in these patients; more importantly, this relationship was observed in those without frailty but not in those with frailty. As non-nephrology physicians and nephrologists are frequently reluctant to prescribe opioids for CKD patients, our findings may assist in the selection of candidates with CKD that can safely receive opioids to achieve satisfactory pain control without excess risk.

## MATERIALS AND METHODS

### Ethical statement

The protocol of the current study adhered to the Declaration of Helsinki, and was approved by the Institutional Review Board of National Taiwan University Hospital (NO. 201802063W). Informed consent was deemed unnecessary due to prior scrambling of patient information with data anonymization.

### Participant identification

Suitable candidates were identified from the Longitudinal Cohort of Diabetes Patients (LCDP), a purposefully assembled cohort consisting of an annual random nationwide sampling of Taiwanese patients with diabetes, between 2004 and 2010. LCDP is frequently utilized for conducting diabetic research in the existing literature [12, 27, 28], with credible findings. In accordance with our study aim, we first confirmed the diagnosis of DM and CKD by imposing the following restrictions: for DM, participants needed to have ≥3 times of diagnosis in outpatient settings or ≥1 time during admission; for CKD, they needed to have validated diagnoses of CKD ≥3 times in outpatient settings or ≥1 time during admission (codes available elsewhere) [27, 29]. In Taiwan, the diagnosis and coding of CKD is usually based on the presence of an estimated glomerular filtration rate (eGFR) < 60 ml/min/1.73m^2^. The day when patients satisfied both diagnoses of DM and CKD was defined as the index date, after which patients started follow-up. Those who were younger than 20 years, with missing data, and those with an inadequate follow-up period (< 1 year; or index date after December 31^st^, 2010) were also excluded. Because opioids are frequently used to achieve satisfactory pain control in those receiving hospice/palliative care [30], and to avoid confounding by indications, we also excluded those who had any reimbursement codes of hospice/palliative care throughout the study period (Figure 1).

We collected demographic profiles (age and sex), lifestyle factors (smoking, alcoholism, and obesity), vital comorbidities, Charlson comorbidity index (CCI) [27] and instrumental treatment variables (coronary revascularization, cardiac surgery, and the receipt of intensive care) before the index date, as well as outcome-influencing medications and anti-diabetic agents between the index date and the end of follow-up. The severity of DM was gauged using the adapted diabetes complications severity index (aDCSI) [31].

### Exposure characterization

The main exposure of this study was the receipt of opioids during the study period. The spectrum of opioids examined consisted of oral and transdermal opioids (buprenorphine, fentanyl, hydromorphone, meperidine, morphine, nalbuphine, oxycodone, propoxyphene, codeine, and tramadol) as single agents or in combination with opioids, excluding those with opioids in cough or flu formulae and methadone (predominantly for addiction treatment in Taiwan), according to prior studies [32, 33]. We divided selected patients into those who received any opioid prescription and those who did not, followed by focusing on those with ≥7 days of opioid exposure within any year and matching them with opioid-naïve ones (throughout the study period) at a 1:1 ratio based on demographic profiles, lifestyle factors, comorbidities and CCI, medications, and treatment variables. Several reasons were responsible for choosing this criterion for defining opioid use in our study. First, a sampling window of one year was selected owing to the concern that opioids are frequently prescribed for the purpose of palliative care among those with a terminal stage of cancer [34], and the scenario is particularly prevalent in Taiwan [35]. If the sampling window is narrow (e.g., within months or even weeks), the probability of recruiting those with a terminal stage of cancer will be high, resulting in selection bias (e.g., opioid users may have more life-threatening diseases than non-users despite matching). Second, prior studies showed that the majority of opioid use commenced within 1 week before or after a given major event [36]. The adoption of a more relaxed criterion (e.g., at least 7 days of use) could be necessary for optimizing sensitivity in the CKD population, since physicians may not feel like prescribing opioids to these patients for fear of side effects [37]. Users who received different types of opioids (each < 7 days) for cumulatively ≥ 7 days were also categorized as cases. Cases (opioid users) and matched controls (opioid-naïve ones) were followed up until death, the end of this study, or December 31^st^, 2011, whichever occurred first.

### Frailty assessment based on the FRAIL scale

We used the renowned FRAIL scale (fatigue, resistance, ambulation, illness, and loss of weight) to detect frailty. Originally created by the International Association of Nutrition and Aging (IANA) [38], the FRAIL scale assesses the biological and functional dimensions of frailty and has been validated as a convenient frailty screening tool among those with DM [39] and CKD [27, 40]. We operationalized the original FRAIL scale based on the diagnosis group combinations outlined elsewhere [12, 27]; briefly, conceptually relevant diagnoses served as proxies for recognizing FRAIL items. For “fatigue,” diagnoses containing keywords of -asthenia, malaise, fatigue, and weakness were identified; for “resistance” stair climbing difficulty was operationalized using diagnoses of debility, fall, and physical deconditioning. The “ambulation” item was operationalized using walking difficulty and gait abnormality, while the “illness” item was recognized based on the original FRAIL definition [38]. Finally, the “weight loss” item was satisfied in the presence of diagnoses related to malnutrition and soft tissue wasting. This modified FRAIL scale has been used repeatedly to study risk factors and adverse health impacts of frailty, with valid results [12, 27, 41]. Frailty was defined as those with ≥ 3 positive FRAIL items, while those with < 3 positive FRAIL items were deemed non-frail [38].

### Statistical analysis

Continuous and categorical variables are described as means ± standard deviations and numbers with percentages in parentheses, respectively, followed by comparisons between groups using Student’s *t*-test and chi-square test, respectively. We first compared demographic profile, lifestyle factors, comorbidities, CCI, diabetic severity, medication usage, and treatment variables between opioid users and propensity score-matched opioid-naïve patients. We also examined the distribution of FRAIL items between users and naïve ones. After follow-up, we examined the influence of opioid use on long-term mortality using Kaplan-Meier survival analysis and Cox proportional hazard regression modeling, incorporating demographic profiles, lifestyle factors, comorbidities, medication use, FRAIL item counts, and treatment variables. The relationship between FRAIL item counts, or frail severity, and the risk of mortality was also examined using Cox proportional hazard regression analyses. Comparisons of survival curves were performed using the log-rank test among the entire cohort and also among groups with and without 1, 2, and >2 FRAIL items. We additionally tested the dose and duration-dependent relationship between opioid exposure and mortality to validate our findings.

We further investigated whether frailty modified the relationship between opioid use and mortality by analyzing the associations between opioid use and mortality in those with frailty, without frailty, without and with 1 and 2 FRAIL items, separately. Two models were created; in model A, we incorporated age/gender, lifestyle factors, all comorbidities, aDCSI, all medications, FRAIL item counts, treatment variables, and opioid use. In model B, we additionally included individual FRAIL item positivity as covariates. Corrections for multiple testing were deemed unnecessary because opioid users/naïve patients in each FRAIL item group were independent from each other. All statistical analyses were performed using STATA version 14 (StataCorp., College Station, TX, USA), and p values < 0.05 were deemed statistically significant.

**Ethics, consent, and permission**

The protocol of the current study was approved by the institutional review board of the National Taiwan University Hospital (NO. 201802063W). Informed consent was waived for all participants as adjudicated by the review board.
